# Economic implications of the different statin prescribing patterns in Central Portugal: a longitudinal analysis

**DOI:** 10.1080/20523211.2026.2614465

**Published:** 2026-01-20

**Authors:** Catarina Abrantes, Luciana G. Negrão, Catarina Coelho, M. Margarida Castel-Branco, Isabel V. Figueiredo, Fernando Fernandez-Llimos

**Affiliations:** a Pharmacology and Pharmaceutical Care Laboratory, Faculty of Pharmacy, University of Coimbra, Coimbra, Portugal; b Laboratory of Pharmacology, Department of Drug Sciences, Faculty of Pharmacy, University of Porto, Porto, Portugal; c Administração Regional de Saúde do Centro, IP (ARSC), Coimbra, Portugal; d Coimbra Institute for Clinical and Biomedical Research (iCBR), Coimbra, Portugal; e Applied Molecular Biosciences Unit (UCIBIO), Porto, Portugal

**Keywords:** Hydroxymethylglutaryl-CoA reductase inhibitors, drug utilisation, longitudinal studies, health services accessibility, drug costs

## Abstract

**Background:**

Statins are one of the most widely used therapeutic classes and have significantly contributed to health care expenditures with reported variability between countries and regions. We aim to identify the economic implications of different statin prescription patterns in the central region of Portugal.

**Methods:**

A retrospective longitudinal study of statin consumption between 2010 and 2022 in the central region, with data obtained from the national administrative claim database. Prescription and expenditure (retail price) were analysed at the municipality level. A score was created for the quartile position (1 = first to 4 = fourth) of each municipality in the distributions of consumption and expenditure for each year. An overall score was created for the study period by aggregating yearly scores. A four-quadrant analysis with the overall scores of cost/DDD and DDD/1000inhabitants/day (DID) was conducted. Bivariate and multivariate analyses were performed.

**Results:**

Statin consumption increased from 64 DDD/1000inhabitants/day (DID) to 149 DID, while cost/DDD and cost/inhabitant decreased from 0.77 € to 0.26 € and from 17.84 € to 13.99 €, respectively. Prescription pattern of high-intensity statins increased from 26% to 74% of the DDD consumed. A four-quadrant plot revealed discrepancies between the municipalities. These discrepancies were associated with the percentage of pitavastatin in DDD (F = 4.604; *p* = 0.005), and the percentage of statins monotherapy in DDD (F = 5.201; *p* = 0.003). Generalising the characteristics of high-level consumption and expenditure municipalities to the entire region would result in a 39.7% increase in expenditure but a 20.8% increase in patients. Prescription of statins per municipality correlated with the prescription of antidepressants (R = 0.643; *p* < 0.001) in the bivariate analysis, and in the multivariate analysis (*p* < 0.001; B = 0.580; 95%CI = 0.416:0.744).

**Conclusion:**

Differences in prescribing patterns resulted in very different proportions of patients treated and expenses associated with the consumption of these lipid-lowering agents. Further analysis should be carried out to understand the financial implications of prescribing new (patent-protected) medicines in Portugal.

## Background

Cardiovascular diseases are the leading cause of death worldwide and in Portugal, with 121.8 deaths per 100 000 population due to ischaemic heart disease, and 117.5 deaths per 100 000 population due to stroke in 2021 (World Health Organization, 2024). Hypercholesterolaemia is a risk factor for cardiovascular events. Statins are the first-line pharmacological treatment for primary prevention of atherosclerotic cardiovascular disease in individuals with multiple risk factors or exhibiting elevated plasma concentrations of low-density lipoprotein cholesterol (LDL-cholesterol; Arnett et al., 2019).

The utilisation of lipid-lowering agents has been continuously increasing in Portugal, with statins accounting for 90% of the total consumption within this therapeutic class in 2013 (Furtado & Oliveira, 2014). In 2023, lipid-lowering drugs emerged as the most widely consumed, ranking fourth in terms of expenditure. In the same year, atorvastatin and rosuvastatin were among the ten most used medicines (Infarmed, 2023).

The market share of generics has also been increasing in Portugal, rising from 10% in 2007 to 30% in 2014 (Gama et al., 2017). This generic increase has been contributing to the reduction of expenditure in medication and causing a decrease in retail prices of non-generic drugs (Bongers & Carradinha, 2009), including statins (Lin et al., 2021). In 2012, simvastatin was the second most used drug for the prevention and treatment of cardiovascular diseases in Portugal, with more than 97% being generics (Gama et al., 2017). Despite the high consumption, simvastatin was not among the highest expenditures in cardiovascular drugs, with the cost of non-generic and generic formulations being very similar (Gama et al., 2017). Conversely, atorvastatin, which had its first generic marketed in 2011, showed an enormous variability in price between generic and brand-name presentations (Gama et al., 2017). The generics rosuvastatin and pitavastatin were marketed in 2017 and 2022, respectively. Gama et al. also demonstrated that the utilisation of generic brands for cardiovascular diseases in 2012 was not optimised, with non-generic medicines and patent-protected statins surpassing the generic statins (Gama et al., 2017). This study concluded that a more rational utilisation of generics could have led to potential cost savings of 275 million euros in Portugal (Gama et al., 2017).

Different consumption patterns of lipid-lowering agents between Portugal and other European countries have been documented (Furtado & Oliveira, 2014; Martinho et al., 2023). A cross-sectional analysis using 2019 data showed that Portugal had an 8.5% utilisation rate of more expensive fixed-dose combinations of statins, while England had 0%, and Denmark or Finland had 0,1% utilisation rate (Martinho et al., 2023). Conversely, the more affordable atorvastatin represented 34.6% in Portugal, but 61.1% in Denmark, 64.3% in Norway, and 69.3% in England (Martinho et al., 2023). Additionally, Portugal was one of the countries with the highest uptake of newer and costly drugs (Martinho et al., 2023). However, this analysis was constrained to a one-year period and utilised aggregated data at the country level, which prevented the detection of localised prescribing patterns. Regional discrepancies in statins consumption have been documented between Portuguese districts (Furtado & Oliveira, 2014) and between the Regional Healthcare Administrations (Rocha et al., 2022), but these analyses lack an economic perspective.

We hypothesised that existing differences in prescribing patterns within this widely utilised therapeutic class, including switching to me-too non-generic drugs, can result in considerable economic consequences and create health care inequalities within Portuguese regions. With this study, we aimed to identify the economic implications of the different statin prescription patterns in the Central region of the country. This work extends the existing evidence by adopting a longitudinal perspective (2010–2022), using both consumption and expenditure data, and incorporating a more granular analysis that includes all municipalities within the Central region.

## Methods

### Setting

Before the modification of the health care geographical structure occurred in January 2024, the Central Region Healthcare Administration (ARS-C) of Portugal was one of the five Regional Health Care Administrations in Portugal mainland. The ARS-C comprised 78 municipalities with a population ranging from 1,7 million inhabitants from 2010 to 2022. In 2022, the ARS-C had 86 health care centres and 548 community pharmacies in a geographical area of 23,670 square kilometres.

Statins are prescription drugs, with the government covering 37% of their reference price. After the prescription is filled by the patient in any community pharmacy, copayment is monthly billed to the Control and Monitoring Center of the National Health Service (NHS) to obtain government reimbursement. Dispensing data is maintained in a centralised database that encompasses the medicines consumed by the Portuguese population and covered by the NHS. Hence, this database is a reliable source of information for drug utilisation studies.

### Data collection

This retrospective longitudinal study was conducted using statin dispensing data of the central region of Portugal (under the administration of the ARS-C), obtained from the NHS centralised database. The dataset provided by the ARS-C on 12/04/2023 included information on all statin prescriptions filled at community pharmacies nationwide, prescribed at ARS-C health care centres from 2010 to 2022. Data were organised by month and by health care centre, and it included for each statin presentation, the quantity of packages dispensed, the dosage, the package size, the total retail price, and the portion reimbursed by the government. No personal identifiers of the users were included in the dataset. The validity and completeness of the data were thoroughly verified. The study was approved by the ARS-C Ethics Committee (reference 28/2022). No personal data or individual patient data or medical records were used in the study. Subsequently, no informed consent was required.

Population was obtained from the PORDATA database (www.pordata.com) on 01/02/2024 as population estimates at 31 December. A monthly estimated population for each municipality was obtained by dividing the difference between consecutive years by the 12 months and adding (or subtracting) the aliquot to the previous month's estimate. For all the year-based analyses, the population estimate corresponding to June was used.

Defined daily doses (DDD) for all the statins (ATC codes C10A, C10B) were obtained from the World Health Organization (WHO) Collaborating Centre for Drug Statistics Methodology (https://atcddd.fhi.no/) on 01/02/2024. To avoid the effects of DDD modifications during the study period, the DDD corresponding to the last year was used (Abrantes et al., 2021). Statins were classified into potential high-intensity statins and potential moderate – or low-intensity statins following the American College of Cardiology/American Heart Association practice guideline (Arnett et al., 2019). Among the seven statins included in this review, only two (i.e. atorvastatin and rosuvastatin) have dosages described as high-intensity, with the remaining five with doses only as low – or moderate-intensity (i.e. fluvastatin, lovastatin, simvastatin, pravastatin, and pitavastatin).

Population density and the population over 65 years old per municipality in 2022 were collected on 01/02/2024 from the National Institute for Statistics (https://www.ine.pt/). Pharmacies and health care centres per municipality in 2022 were extracted from the Portuguese Medicines Agency (https://www.infarmed.pt/web/infarmed) and the Central Administration of the Health System (https://www.acss.min-saude.pt/) on 01/02/2024, respectively. Antidepressant consumption (N06A) data expressed in DDDs per 1000 inhabitants per day (DDD/1000inh/day) was obtained from a previous study (Negrão et al., 2024).

### Data analysis

Statin consumption was expressed in DDD, calculated according to the following equation:

$$\mathrm{DDD}=\frac{\mathrm{Dosage}*\mathrm{Number}\;\mathrm{of}\;\mathrm{tablets}\;\mathrm{per}\;\mathrm{package}*\mathrm{Number}\;\mathrm{of}\;\mathrm{packages}}{\mathrm{DDD}(\mathrm{WHO})}$$

In the case of fixed-dose combinations, DDD of the statin portion was used.

Yearly expenditure on retail prices and prescribed DDDs was obtained by the summation of the corresponding monthly values. For each geographical area (municipalities and ARS-C), the number of DDD/1000inh/day was calculated for each year considering the DDDs prescribed, the population and 365 days as the period, using the following equation:

$$\mathrm{DDD}/1000\mathrm{inh}/\mathrm{day}=\frac{\mathrm{DDD}}{\;\mathrm{population}*365}*1000$$

Overall DDD/1000inh/day for the entire study period was calculated using the total DDD consumed during the study period, considering 4,745 days (365 days*13 years).

Variability of the metrics across municipalities (i.e. DDD/1000inh/day, cost per DDD, and cost per inhabitant) was calculated by the percentage of the interquartile range (IQR) out of the median. For each year, the municipality position in the quartile distribution of these metrics was calculated and a score from 1 (i.e. first quartile, <25%) to 4 (i.e. fourth quartile, >75%). The quartile position score provides a rank-based measure of relative position, thus enabling the classification of each municipality according to its standing within the distribution for each year. To obtain a metric that embraces the quartile position for each of these metrics during the entire study period, a mean of the quartile position scores of each year was calculated for each municipality. Hence, a synthetic longitudinal metric was obtained, which summarises persistent patterns rather than isolated annual fluctuations. This approach enables the identification of municipalities with systematically higher or lower prescribing or cost levels.

The mean of the quartile position scores of antidepressants (N06A) consumption was also calculated. The utilisation of antidepressant consumption data from a preceding study (Negrão et al., 2024) enabled the establishment of a comparator group. Antidepressant drugs have a potential target population with different characteristics than antidyslipidemic users, which allows for exploring discrepancies in prescribing patterns originating from different reasons related to epidemiological patient characteristics. Therefore, in the present work, the use of antidepressants ensured methodological consistency and provided a relevant benchmark within primary care prescribing.

A four-quadrant plot was created with the mean of the quartile position scores for DDD/1000inh/day and the mean of the quartile position scores for cost per DDD, establishing the coordinates 2.5,2.5 as the origin of the quadrant. The distance of each municipality to the origin was calculated using the Pythagorean theorem.

$$\mathrm{Distance}=\sqrt{{\left(\mathrm{DDD}1000\mathrm{Dscore}-2.5\right)}^{2}+{\left(\cos t_{\mathrm{DDD}}\mathrm{score}-2.5\right)}^{2}}$$

This method allows a simultaneous analysis of the utilisation intensity and expenditure, categorising municipalities into four quadrants: Quadrant 1 – High prescription, high cost; Quadrant 2 – High prescription, low cost; Quadrant 3 – Low prescription, low cost; and Quadrant 4 – Low prescription, high cost.

To conduct a simulation of the budget and epidemiological impact of generalising each of the four quadrants' characteristics, a similar analysis (the four quadrants analysis) was conducted using 2022 raw data. Overall, ARS-C statin expenditure and prescription intensity were calculated for four scenarios created with the median DDD/1000inh/day and the median cost per DDD of each of the four quadrants.

Descriptive statistics were performed, presenting the metrics as median and IQR to classify the municipalities into four groups. Normality was explored with the Kolmogorov–Smirnov test with additional inspection of the quintile-quintile plot. Characteristics of the municipalities falling into each of the four quadrants were compared through an ANOVA. Pearson’s correlation analyses were conducted between quartile position scores and several socio-demographic covariates. Also, a multivariate linear regression was conducted to identify the association between quartile position scores for yearly distributions of DDD/1000inh/day and these covariates. All the statistics were conducted using SPSS v.28 (IBM, Armonk, NY, United States) establishing a significance limit of 0.05.

## Results

During the 13 years of analysis, the 803 million DDD of statins consumed in ARS-C had a total cost of 288 million euros, with a 41.3% portion (119 million) covered by the government (Table 1). A positive trend existed for the statin consumption, from the initial 64 DDD/1000inh/day in 2010 to the 149 DDD/1000inh/day in 2022 (y = 6.4x + 55.3; R^2^ = 0.978). However, expenditure evolution presented a different shape, with a drastic decrease from 31 million euros in 2010–21 million in 2012, remaining quite constant until 2022. Cost per DDD suffered a rapid decrease from 2010 to 2013 and then a slow decrease until 2022. The combination of DDD increase and cost decrease resulted in a cost per inhabitant decrease in 2011, but a slight increase from 2012 to 2022 (Table 1).

**Table 1.** Consumption of statins in the central region of Portugal (ARS-C).

Year	Total cost	Government share	DDD	DDD/1000inh/day	Cost per DDD	Cost per inh
2010	31,234,047	17,670,368	40,561,007	63.47	0.77	17.84
2011	25,798,050	11,350,944	44,111,269	69.42	0.58	14.82
2012	20,923,120	9,017,771	48,418,355	76.80	0.43	12.11
2013	19,558,778	7,614,936	51,976,065	83.14	0.38	11.42
2014	20,302,774	7,794,298	54,832,515	88.43	0.37	11.95
2015	21,044,148	8,045,176	57,765,163	93.72	0.36	12.46
2016	21,008,046	7,897,103	59,999,057	97.85	0.35	12.50
2017	21,443,394	8,418,339	62,810,016	103.09	0.34	12.85
2018	19,765,399	7,314,113	65,671,884	108.54	0.30	11.92
2019	20,188,145	7,713,452	69,994,847	116.16	0.29	12.23
2020	21,438,868	8,240,793	75,520,434	125.08	0.28	12.96
2021	22,374,242	8,709,419	81,640,013	134.64	0.27	13.47
2022	23,351,505	9,392,674	90,661,586	148.82	0.26	13.99

DDD: Defined daily dose; inh: inhabitants.

**Figure 1.**
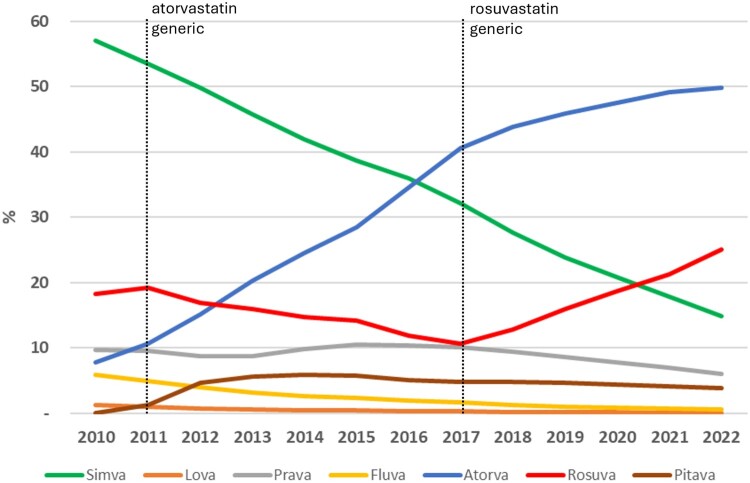
Statin consumption in percentage of overall defined daily dose (DDD) in central region of Portugal (ARS-C).

**Figure 2.**
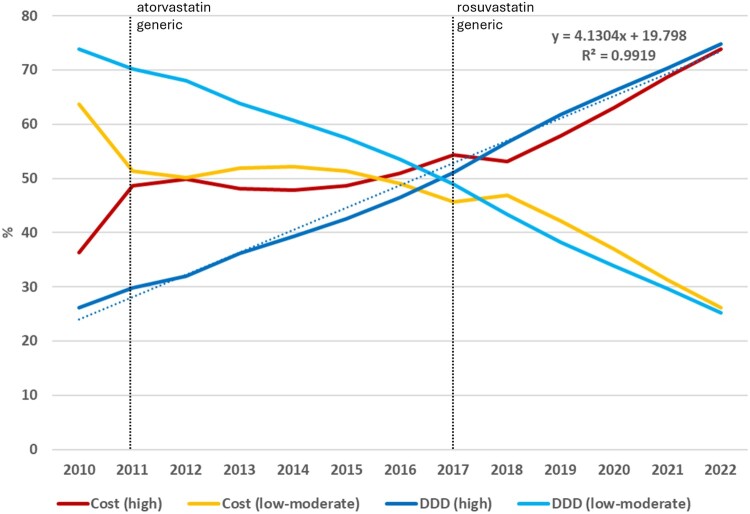
Trends of market share in cost (cost per DDD) and prescription (DDD) of high and low-moderate intensity statins for central region of Portugal (ARS-C).

Three of the seven statins had a relevant consumption with different patterns in the ARS-C during the study period. Simvastatin suffered a continuous decrease from 57% to 15%, while atorvastatin increased from 7% to 50%. Rosuvastatin presented two different trends, decreasing from 2011 to 2017, and increasing then until 2022 (Figure 1). A positive trend of the percentage of DDD consumed of high-intensity statins (atorvastatin and rosuvastatin) was evident (y = 4.13x + 19.8; R^2^ = 0.992), starting from 26% in 2010 until 74% in 2022. A non-parallel trend of the percentage of expenditure of high-intensity statins existed until 2018 (Figure 2).

A variability in the DDD/1000inh/day, in the cost per DDD, as well as in the cost per inhabitant was observed across municipalities in all the years (Table 2). A wider variability was observed for the cost per inhabitant, with the IQR varying from 21.5% of the median in 2011–29.2% in 2020, while the IQR of the cost per DDD varied from 8.3% of the median in 2010–18.9% in 2017. This different range of the two metrics was originated by the wide variability of the DDD/1000inh/day across municipalities, which varied from 21.9% of the median in 2012–28.6% in 2022.

**Table 2.** Variability of DDD/1000inhabitans/day and cost per DDD across the 78 municipalities of the central region of Portugal (ARS-C).

Year	DDD/1000inh/day		Cost per DDD (EUR)		Cost per inhabitant (EUR)	
	Median	IQR	Median	IQR	Median	IQR
2010	71.3	60.6: 77.0	0.76	0.74: 0.80	19.9	16.7: 21.1
2011	76.0	65.5: 83.8	0.58	0.54: 0.61	15.9	14.1: 17.5
2012	84.2	74.3: 92.7	0.42	0.39: 0.46	12.9	11.4: 14.9
2013	92.3	78.7: 101.4	0.37	0.34: 0.41	12.2	10.8: 13.9
2014	96.5	84.5: 108.7	0.37	0.34: 0.40	12.5	10.9: 15.1
2015	100.2	87.1: 114.4	0.36	0.34: 0.40	13.1	11.7: 15.5
2016	104.4	91.3: 118.9	0.35	0.32: 0.38	13.3	11.6: 15.3
2017	109.2	95.6: 123.3	0.34	0.32: 0.38	13.6	11.9: 15.8
2018	115.0	101.5: 129.6	0.30	0.28: 0.33	12.9	10.9: 14.4
2019	123.0	109.6: 139.7	0.29	0.27: 0.32	13.3	11.4: 15.2
2020	130.9	119.5: 152.6	0.29	0.27: 0.32	14.2	11.8: 16.0
2021	140.7	124.9: 160.3	0.28	0.26: 0.30	14.7	12.4: 16.2
2022	152.7	136.6: 180.3	0.28	0.25: 0.30	15.9	13.6: 18.2

DDD: Defined daily dose; IQR: interquartile range (represented by 25% and 75% quartiles); inh: inhabitants.

The mean of the quartile position scores for yearly distributions of DDD/1000inh/day presented a weak correlation with population density (R = −0.267; *p* = 0.018), percentage of population over 65 years (R = 0.319; *p* = 0.004), population per pharmacy (R = −0.262; *p* = 0.021), population per health care centre (R = −0.275; *p* = 0.015), but a moderate correlation with the mean of the quartile position scores of antidepressants (N06A) of a previous study (R = 0.643; *p* < 0.001). The mean of the quartile position scores for yearly distributions of cost per DDD presented weak correlations only with percentage of population over 65 years (R = 0.331; *p* = 0.003) and the mean of the quartile position scores of antidepressants (R = −0.318; *p* = 0.005). In a multivariate analysis for the mean of the quartile position scores for yearly distributions of DDD/1000inh/day as dependent variable with all these covariates, only the mean of the quartile position scores of antidepressants showed significant association (*p* < 0.001; B = 0.580; 95%CI = 0.416:0.744; R2 = 0.501; Durbin-Watson = 1.640).

**Figure 3.**
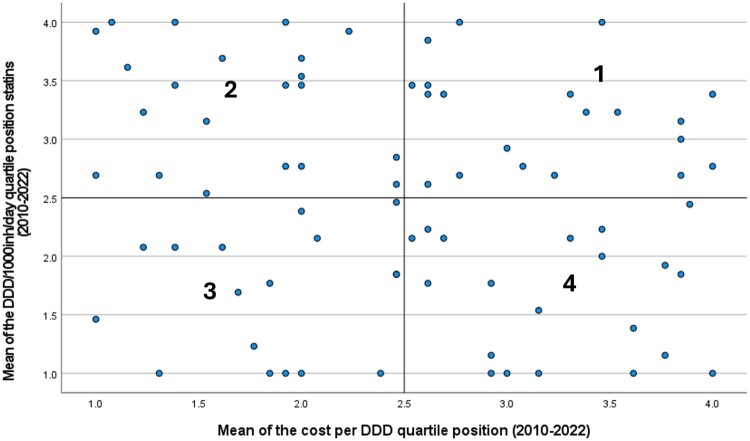
Four-quadrant analysis with the means of the quartile position scores for yearly distributions of DDD/1000inh/day and cost per DDD of statins in central region of Portugal (ARS-C). DDD: Defined daily dose; inh: inhabitants.

In the four-quadrant plot (Figure 3), 20 municipalities fell in the first quadrant (high prescription, high cost), 20 in the second (high prescription, low cost), 17 in the third (low prescription, low cost), and 21 in the fourth (low prescription, high cost). Table 3 compiles the different characteristics of municipalities included in each of the four quadrants (municipality-level details in Supplemental file 1). Significant association was identified between municipalities in the four quadrants and DDD/1000inh/day statins 2010–2022 (F = 31.339; *p* < 0.001), cost per DDD (EUR) 2010–2022 (F = 27.214; *p* < 0.001), % pitavastatin in DDD 2010–2022 (F = 4.604; *p* = 0.005), and % statins monotherapy in DDD 2010–2022 (F = 5.201; *p* = 0.003), but not with % atorvastatin in DDD 2010–2022 (F = 1.981; *p* = 0.124), % rosuvastatin in DDD 2010–2022 (F = 2.594; *p* = 0.059).

**Table 3.** Characteristics of the municipalities in the four quadrants of the mean of the quartile position scores for DDD/1000inh/day and cost per DDD.

	First quadrant High prescription High cost		Second quadrant High prescription Low cost		Third quadrant Low prescription Low cost		Fourth quadrant Low prescription High cost	
	Median	IQR	Median	IQR	Median	IQR	Median	IQR
DDD/1000inh/day statins 2010–2022	118.3	109.5: 124.8	119.5	110.5: 128.7	97.5	86.0: 102.2	96.9	78.5: 100.4
Cost per DDD (EUR) 2010–2022	0.39	0.37: 0.43	0.34	0.32: 0.35	0.35	0.32: 0.36	0.39	0.37: 0.40
% Atorvastatin in DDD 2010–2022	35.3	22.2: 41.4	38.6	33.0: 41.8	34.6	30.4: 40.7	31.2	24.4: 39.2
% Rosuvastatin in DDD 2010–2022	17.6	15.3: 22.6	14.5	11.7: 18.0	17.3	15.0: 18.4	17.4	13.9: 21.0
% Pitavastatin in DDD 2010–2022	5.5	3.1: 6.4	3.1	2.3: 4.2	3.9	2.6: 4.9	4.3	3.6: 5.5
% Statin monotherapy in DDD 2010–2022	92.3	87.7: 92.3	94.9	91.2: 94.9	92.8	89.8: 92.8	92.6	87.1: 92.6

DDD: Defined daily dose; inh: inhabitants.

The scenarios created with the simulations by generalising the metrics of each of the quadrants to the entire ARS-C are presented in Table 4. In quadrants 3 and 4, the lower DDD/1000inh/day resulted in fewer patients (estimated) treated with statins. But only in quadrant 3 did this lower prescription produce a lower expenditure, due to the higher cost per DDD of quadrant 4. Generalising the characteristics of quadrant 1, high prescription and high cost, to the entire ARS-C would result in a 39.7% increase in expenditure but only a 20.8% increase in patients treated.

**Table 4.** Simulation of the effects of generalising the characteristics of each of the quadrants to all the ARS-C.

	DDD/1000inh/day	Cost per DDD	Patients Treated*		Expenditure	
	(median)	(median)	(number)	% Difference**	(EUR)	% Difference**
Actual	152.7	0.26	255,172	–	23,351,505	–
Quadrant 1	184.4	0.29	308,144	+20.8	32,617,083	+39.7
Quadrant 2	174.6	0.25	291,768	+14.3	26,623,825	+14.0
Quadrant 3	142.8	0.26	238,628	−6.5	22,645,805	−3.0
Quadrant 4	133.3	0.31	222,753	−12.7	25,204,498	+7.9

Quadrant 1 = high prescription, high cost; Quadrant 2 = high prescription, low cost; Quadrant 3 = low prescription, low cost; Quadrant 4 = low prescription, high cost.

* Estimated using the DDD/1000inh/day and the population (1,671,065 inhabitants).

** Difference between the actual expenditure and the estimated number of patients treated with the actual DDD prescribed.

DDD: Defined daily dose; inh: inhabitants.

### Discussion

This longitudinal study described the statins prescribing patterns in the ARS-C municipalities for a period of 13 years. The consumption of statins exhibited an increasing trend, having more than doubled throughout the period studied. The utilisation pattern of low-moderate and high-intensity statins inverted, with the consumption of low-moderate statins substantially decreasing, while the high-intensity statins increased. Important discrepancies between the 78 municipalities were found, related to the levels of consumption of these lipid-lowering drugs, as well as in the expenditure associated with the utilisation of this therapeutic class. The adoption of the different patterns by different municipalities would result in very different levels of consumption and expenditure.

The increasing use of statins described in our study was also reported in several countries around the world (Blais et al., 2021; Ofori-Asenso et al., 2018), including in Europe (O’Keeffe et al., 2016; Reinau et al., 2021). This growing trend may be related to an increase in the prevalence of cardiovascular diseases and associated risk factors (Bourbon et al., 2019), the aging of the population, changes in clinical guidelines (Matyori et al., 2023), pharmaceutical industry marketing (Fickweiler et al., 2017) or disease-mongering (González-Moreno et al., 2015). Additionally, the reduction in expenditure associated with the use of generic statins (Gama et al., 2017) may also influence adherence and consumption levels. However, one study conducted in Australia reported a decreasing trend between 2013-2019, despite statins remaining the most prevalent lipid-lowering agents prescribed. The authors stated that this decrease may be related to concerns about adverse events, the increase in the utilisation of non-statin therapies, or economic factors related to the Australian reimbursement mechanisms (Talic et al., 2022).

Contrary to the recommendations of Portuguese guidelines (Macedo et al., 2017), we observed a decreasing trend in the prescription of simvastatin, with an increase in atorvastatin and rosuvastatin, which are the only statins that could achieve high-intensity dosages. These prescription patterns may be a reaction to international guidelines that recommend the use of high-intensity statins in patients with diabetes and multiple cardiovascular disease risk factors, as well as in adults with an LDL cholesterol over 190 mg/dL (Arnett et al., 2019). Other countries have also adopted these recommendations, resulting in a rise in the utilisation of high-intensity statins (Lin et al., 2021; Ofori-Asenso et al., 2018; Reinau et al., 2021).

It is important to note that the prescription of rosuvastatin decreased in 2011, concurrently accompanied by a rise in the use of atorvastatin, which became available in its generic form during that year. The decreasing trend for rosuvastatin stopped in 2017 with the introduction of generic rosuvastatin. This indicates that physicians in the central region demonstrated a strong inclination towards generics, favouring the prescription of more affordable high-intensity statins. The alterations in prescription practices have contributed to the reduction of cost per DDD over the years, as well as to changes in the trend regarding the percentage of expenditure of statins with different intensity. Moreover, although data on adherence were lacking, the introduction of generic statins may have facilitated patient access and influenced treatment patterns. The strong decline in overall expenditure observed during the initial three years of the study could be attributed to the revision of medicines pricing policies that occurred in 2010, which led to a reduction of 35% in retail prices of generics (Diário da República, 2010). Previous studies highlighted the disparity in statin consumption between Portugal and other European countries (Furtado & Oliveira, 2014; Gama et al., 2017; Martinho et al., 2023), which may be attributed to the implementation of several guidelines across different countries. However, our study further revealed that such discrepancies are also present among Portuguese municipalities within the same regional administration, resulting in different levels of statin consumption and associated expenditures. Additionally, the identified discrepancies did not correlate with socio-demographic variables, suggesting that epidemiological associations are not a determinant driver of these geographical variations. Conversely, a correlation with intensive prescription patterns for other classes was found. Despite Zhang et al. (2022) and Gutlapalli et al. (2022) showing that cardiovascular disease and mental disorders can co-occur, we selected antidepressants as a comparator because they have different target patient profiles. The strong correlation between the prescription intensity of these two classes across municipalities may point to a potential influence of prescribing behaviours. Hence, it is important to conduct further analyses involving different therapeutic classes to determine if these associations persist.

The four-quadrant analysis revealed uneven associations between the overall consumption of statins and its associated expenditures, which demonstrates the discriminant power of this analysis. This analysis also showed that the disparities in expenditure within the central region were not attributable to the prescription of high-intensity statins. Instead, these discrepancies are associated with varying patterns in the prescription of pitavastatin, a moderate-intensity statin that held patent protection during the study period, as well as fixed-dose combinations. This is in line with the findings of previous studies that identified Portugal as a country with a high adoption of new and expensive medicines (Furtado & Oliveira, 2014; Martinho et al., 2023). Systematic reviews have described several factors influencing the uptake of new medicines (Lublóy, 2014; Medlinskiene et al., 2021). Among the medicine-related factors, the high cost of a new drug emerged as a barrier, albeit to varying degrees. Some prescribers may overlook the cost (Medlinskiene et al., 2021), prioritising safety and perceived efficacy instead (Lublóy, 2014). New medications regarded as therapeutically innovative tended to be adopted more rapidly (Lublóy, 2014; Medlinskiene et al., 2021). Prescribing patterns serve as strong predictors of new drug uptake, with high prescribing volumes or wider prescribing portfolio increasing the likelihood of early adoption of new medicines (Lublóy, 2014). Likewise, pharmaceutical detailing and industry were also identified as important factors (Lublóy, 2014; Medlinskiene et al., 2021), with evidence indicating that stringent policies limiting pharmaceutical detailing led to a delay in the adoption of new medicines (Medlinskiene et al., 2021). Hence, the prescription of expensive medications underscores the necessity for additional studies regarding the financial implications of innovation in Portugal and elsewhere.

In this study, different prescription patterns were evident across municipalities, mainly originating from discrepancies in the prescription of higher-priced statins, which resulted in very different intensities of prescription and different costs per dose prescribed. Four simulation exercises gave an insight into the economic and epidemiological consequences of generalising each of the four prescribing patterns to the entire region. Generalising the pattern of municipalities with high prescription intensity and high cost (first quadrant) would result in a rise in the number of patients receiving statin treatment of about 20% but of 40% in the cost of their treatment. Also, generalising the prescription pattern of low-intensity and high-cost municipalities (fourth quadrant) would lead to an undesirable situation with an increase in the cost of 7% while reducing the number of patients treated by 13%. Alternatively, adopting for the entire region the prescription patterns of the high-prescription intensity and low-cost municipalities (second quadrant) would result in an increase, both in the cost and the number of patients treated of 14%. It seems that promoting the prescription of low-cost (i.e. patent-expired and generic-available) statins would allow for an increase in the proportion of patients treated with no excessive expenditure associated.

### Strengths and limitations

Using real-world data, obtained from a reliable national database that encompasses all the population, we were able to describe the prescribing patterns of statins and their associated expenditures in the central region of Portugal. Also, the higher granularity of our analysis enabled us to identify geographic discrepancies in consumption and expenditure levels between municipalities. This design allowed for a more comprehensive understanding of temporal and spatial variations in statin use and their associated economic impact. However, some limitations existed for this study. We classified atorvastatin and rosuvastatin as high-intensity statins, even though they can also be prescribed in moderate intensity doses. Given the administrative nature of the dataset, we had no access to the actual dosages prescribed to patients, so the proportion of patients on high-intensity statins may be overestimated, but not their costs. This analysis is unable to identify which municipalities exhibit ideal prescription patterns, as an investigation of related health outcomes, namely LDL-cholesterol plasma concentrations, cardiovascular events or related hospitalisations, is necessary for such an assessment. Future research should be conducted to assess which municipalities achieved optimal clinical outcomes, thus determining the influence of prescribing patterns on patient health outcomes and their relevance to public health. Nonetheless, our findings allowed for the identification of potential inefficiencies or inequalities between municipalities, which may indirectly impact population health.

Additionally, this study focused on outpatient statin consumption and expenditure. In Portugal, statins are prescribed by family physicians to community dwelling patients, and only statins used during patients' hospitalisation periods would be prescribed by hospital physicians, which represents a negligible proportion of total statin consumption.

## Conclusions

Our study shows that the consumption of statins in the central region of Portugal has been increasing, with the reversal of prescription patterns of low-moderate intensity statins with high-intensity ones. Important discrepancies existed among the municipalities of the central region, resulting in very different proportions of patients treated with statins, as well as in the cost per dose of these medicines. Differences in these patterns would result in very different levels of expenditure. The existing variations in prescription were associated with high levels of prescription of other therapeutic classes, with the prescription of pitavastatin and fixed-dose combinations, revealing different prescribing profiles among the municipalities. Our analysis provides important information that could allow the development of strategies to enhance the rational prescription of statins, leading to cost savings that contribute to the sustainability of the Portuguese healthcare system. Additional analyses are necessary to confirm the existence of associations between high prescription patterns, as well as to understand the financial implications of innovation in Portugal.

## Supplementary Material

Supplemental Material

## Data Availability

The datasets generated and analysed during the current study are not publicly available because they were provided in a restricted form by the Healthcare Authorities. Researchers interested in the original datasets should request permission from ARS-C.

## Abbreviations

ANOVA: analysis of variance; ARS: Regional Healthcare Administration; ATC: Anatomical Therapeutic Chemical classification; CoA: coenzyme A; DDD: defined daily dose; DID: DDD per 1000 inhabitants and day; Inh: inhabitants; IQR: interquartile range; LDL: low density lipoprotein; NHS: National Health Service; WHO: World Health Organization.

## References

[CIT0001] Abrantes, C., Tonin, F. S., Reis-Pardal, J., Castel-Branco, M., Furtado, C., Figueiredo, I. V., & Fernandez-Llimos, F. (2021 September). Implications of a defined daily dose fixed database for drug utilization research studies: The case of statins in Portugal. *British Journal of Clinical Pharmacology*, *87*(9), 3542–3549. 10.1111/bcp.14770 33576512

[CIT0002] Arnett, D. K., Blumenthal, R. S., Albert, M. A., Buroker, A. B., Goldberger, Z. D., Hahn, E. J., Himmelfarb, C. D., Khera, A., Lloyd-Jones, D., William McEvoy, J., Michos, E. D., Miedema, M. D., Muñoz, D., Smith, S. C., Virani, S. S., Williams, K. A., Yeboah, J., & Ziaeian, B. (2019, September 10). Acc/AHA guideline on the primary prevention of cardiovascular disease: A report of the American College of Cardiology/American Heart Association task force on clinical practice guidelines. *Circulation*, *140*(11), e596–e646. 30879355 10.1161/CIR.0000000000000678 PMC7734661

[CIT0003] Blais, J. E., Wei, Y., Yap, K. K. W., Alwafi, H., Ma, T.-T., Brauer, R., Lau, W. C. Y., Man, K. K. C., Siu, C. W., Tan, K. C. B., Wong, I. C. K., Wei, L., & Chan, E. W. (2021 July). Trends in lipid-modifying agent use in 83 countries. *Atherosclerosis*, *328*, 44–51. 10.1016/j.atherosclerosis.2021.05.016 34091069

[CIT0004] Bongers, F., & Carradinha, H. (2009 June). How to increase patient access to generic medicines in European healthcare systems: European Generic Medicines Association.

[CIT0005] Bourbon, M., Alves, A. C., & Rato, Q. (2019). Prevalence of cardiovascular risk factors in the Portuguese population. Instituto Nacional de Saúde Doutor Ricardo Jorge (INSA, IP), Lisbon.

[CIT0006] Diário da República. (2010). 1st series, N.^o^ 112, June 11, 2010. [Ordinance No. 312-A/2010, of June 11] [Internet]. https://diariodarepublica.pt/dr/home

[CIT0007] Fickweiler, F., Fickweiler, W., & Urbach, E. (2017 September). Interactions between physicians and the pharmaceutical industry generally and sales representatives specifically and their association with physicians’ attitudes and prescribing habits: A systematic review. *BMJ Open*, *7*(9), e016408. 10.1136/bmjopen-2017-016408 PMC5623540 28963287

[CIT0008] Furtado, C., & Oliveira, R. (2014). Statins 2000-2013: Utilization, expenditure, regional and international variations. Lisboa: Gabinete de Informação e Planeamento Estratégico, Infarmed I.P., Lisbon.

[CIT0009] Gama, H., Torre, C., Guerreiro, J. P., Azevedo, A., Costa, S., & Lunet, N. (2017). Use of generic and essential medicines for prevention and treatment of cardiovascular diseases in Portugal. *BMC Health Services Research*, *17*(1), 449. 10.1186/s12913-017-2401-2 28662649 PMC5492988

[CIT0010] González-Moreno, M., Saborido, C., & Teira, D. (2015 June). Disease-mongering through clinical trials. *Studies in History and Philosophy of Science Part C: Studies in History and Philosophy of Biological and Biomedical Sciences*, *51*, 11–18. 10.1016/j.shpsc.2015.02.007 25863220

[CIT0011] Gutlapalli, S., Chaudhuri, D., Khan, K., Al Shouli, R., Allakky, A., Ferguson, A. A., Khan, A. I., Abuzainah, B., & Mohammed, L. (2022, December 8). Statins and antidepressants: A comprehensive review and clinical outlook of the risks and benefits of co-prescription. *Cureus*, *14*(12), e32331. 36632257 10.7759/cureus.32331 PMC9827898

[CIT0012] Infarmed, I. P. (2023 December). Analysis of medication consumption in outpatient settings – December 2023 [Internet]. Lisbon: INFARMED - Autoridade Nacional do Medicamento e Produtos de Saúde, I.P. https://www.infarmed.pt/web/infarmed

[CIT0013] Lin, S. y., Baumann, K., Zhou, C., Zhou, W., Cuellar, A. E., & Xue, H. (2021, November 22). Trends in Use and expenditures for brand-name statins after introduction of generic statins in the US, 2002–2018. *JAMA Network Open*, *4*(11), e2135371. 10.1001/jamanetworkopen.2021.35371 34807258 PMC8609409

[CIT0014] Lublóy, Á. (2014 December). Factors affecting the uptake of new medicines: A systematic literature review. *BMC Health Services Research*, *14*(1), 469. 10.1186/1472-6963-14-469 25331607 PMC4283087

[CIT0015] Macedo, M. D., Gonçalves, C., Silva, A. E., Canhota, C., Rocha, E., Silva, J. M., et al. (2017, May 11). Standard n.o 019/2011: Therapeutic approach to dyslipidemia in adults. DGS, Lisbon.

[CIT0016] Martinho, I., Teixeira Rodrigues, A., Guerreiro, J., Rocha, J., Sepodes, B., & Torre, C. (2023, January 2). A cross-country utilization patterns comparison of high expenditure therapeutic groups between Portugal and six European countries: The two sides of the Portuguese coin. *Expert Review of Pharmacoeconomics & Outcomes Research*, *23*(1), 89–97. 10.1080/14737167.2023.2144839 36345962

[CIT0017] Matyori, A., Brown, C. P., Ali, A., & Sherbeny, F. (2023 June). Statins utilization trends and expenditures in the U.S. before and after the implementation of the 2013 ACC/AHA guidelines. *Saudi Pharmaceutical Journal*, *31*(6), 795–800. 10.1016/j.jsps.2023.04.002 37228328 PMC10203693

[CIT0018] Medlinskiene, K., Tomlinson, J., Marques, I., Richardson, S., Stirling, K., & Petty, D. (2021 December). Barriers and facilitators to the uptake of new medicines into clinical practice: A systematic review. *BMC Health Services Research*, *21*(1), 1198. 10.1186/s12913-021-07196-4 34740338 PMC8570007

[CIT0019] Negrão, L. G., Coelho, C., Castel-Branco, M. M., Figueiredo, I. V., & Fernandez-Llimos, F. (2024). Impact of the COVID-19 pandemic on antidepressant consumption in the central region of Portugal: Interrupted time series. *Social Psychiatry and Psychiatric Epidemiology*, *60*(3), 621–629. 39001886 10.1007/s00127-024-02731-0 PMC11870879

[CIT0020] Ofori-Asenso, R., Ilomäki, J., Zomer, E., Curtis, A. J., Zoungas, S., & Liew, D. (2018 June). A 10-year trend in statin Use among older adults in Australia: An analysis using national pharmacy claims data. *Cardiovascular Drugs and Therapy*, *32*(3), 265–272. 10.1007/s10557-018-6794-x 29790056

[CIT0021] O’Keeffe, A., Nazareth, I., & Petersen, I. (2016 May). Time trends in the prescription of statins for the primary prevention of cardiovascular disease in the United Kingdom: A cohort study using the health improvement network primary care data. *Clinical Epidemiology*, *27*(8), 123–132. 10.2147/CLEP.S104258 PMC4890684 27313477

[CIT0022] Reinau, D., Schur, N., Twerenbold, S., Blozik, E., Früh, M., Signorell, A., Schwenkglenks, M., & Meier, C. R. (2021, September 1). Utilisation patterns and costs of lipid-lowering drugs in Switzerland 2013–2019. *Swiss Medical Weekly*, *151*(3536), w30018. 10.4414/SMW.2021.w30018 34495601

[CIT0023] Rocha, V., Plácido, A. I., Rodrigues, D. A., Tavares, A. B., Figueiras, A., Roque, F., & Herdeiro, M. T. (2022, October 10). Geographic variation in Top-10 prescribed medicines and potentially inappropriate medication in Portugal: An ecological study of 2.2 million older adults. *International Journal of Environmental Research and Public Health*, *19*(19), 12938. 10.3390/ijerph191912938 36232238 PMC9564588

[CIT0024] Talic, S., Marquina Hernandez, C., Ofori-Asenso, R., Liew, D., Owen, A., Petrova, M., Lybrand, S., Thomson, D., Ilomaki, J., Ademi, Z., & Zomer, E. (2022 July). Trends in the utilization of lipid-lowering medications in Australia: An analysis of national pharmacy claims data. *Current Problems in Cardiology*, *47*(7), 100880. 10.1016/j.cpcardiol.2021.100880 34108083

[CIT0025] World Health Organization. (2024, December 6). Health data overview for the Portuguese republic [Internet]. https://data.who.int/countries/620.

[CIT0026] Zhang, L., Bao, Y., Tao, S., Zhao, Y., & Liu, M. (2022 January). The association between cardiovascular drugs and depression/anxiety in patients with cardiovascular disease: A meta-analysis. *Pharmacological Research*, *175*, 106024. 10.1016/j.phrs.2021.106024 34890773

