# Supplementary material for: Economic implications of the different statin prescribing patterns in Central Portugal: a longitudinal analysis
Source: J Pharm Policy Pract. 2026 Jan 20;19(1):2614465. doi: 10.1080/20523211.2026.2614465 (PMC12821345; doi:10.1080/20523211.2026.2614465)
Supplement: Supplemental Material [file JPPP_A_2614465_SM7049.pdf]

Economic implications of the different statin prescribing patterns in Central Portugal: a longitudinal analysis.

Supplementary files

Supplementary File 1. Municipality position in the four-quadrant analysis.

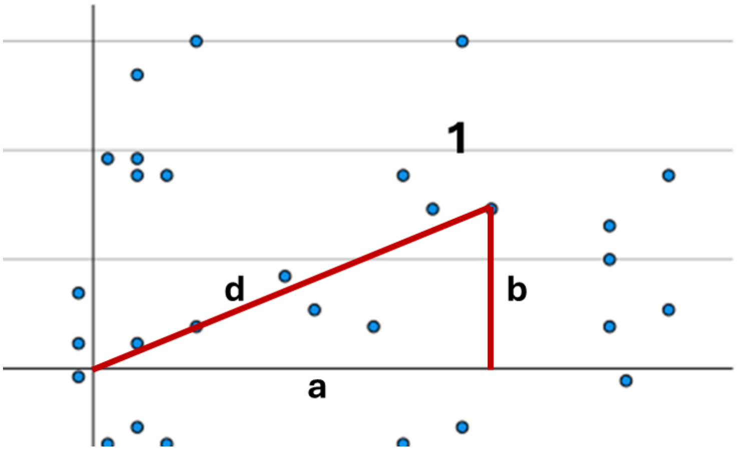

$$d = \sqrt{(a - 2.5)^2 + (b - 2.5)^2}$$

d=distance

a= mean of the quartiles of the cost per DDD yearly distributions

b= mean of the quartiles of the DDD/1000inh/day yearly distributions

Municipality	Mean of the quartiles of the yearly distributions		quad-rant	distance	Antidepressants mean of the quartiles of the yearly distributions (DDD/1000inh/day)
	DDD/1000inh/day	cost per DDD			
Quadrant 1<br>High prescription<br>High cost	Quadrant 2<br>High prescription<br>Low cost		Quadrant 3<br>Low prescription<br>Low cost		Quadrant 4<br>Low prescription<br>High cost
Águeda	2.15	3.31	4	0.879	2.42
Aguiar da Beira	3.23	3.54	1	1.270	3.17
Albergaria-a-Velha	1.85	2.46	3	0.655	2.33
Almeida	2.77	3.08	1	0.637	1.17
Alvaiázere	2.38	2.00	3	0.513	3.50
Anadia	3.46	1.38	2	1.473	2.67
Ansião	3.54	2.00	2	1.153	3.50
Arganil	2.62	2.62	1	0.163	1.42
Aveiro	1.15	2.92	4	1.411	1.83
Batalha	3.15	1.54	2	1.163	3.08
Belmonte	1.85	2.46	3	0.655	1.92
Cantanhede	3.62	1.15	2	1.748	2.92
Carregal do Sal	4.00	1.92	2	1.607	3.67
Castanheira de Pera	3.38	2.62	1	0.892	3.92
Castelo Branco	1.00	3.62	4	1.869	2.00
Castro Daire	2.69	1.00	2	1.512	2.08
Celorico Beira	1.77	1.85	3	0.981	1.08
COIMBRA	2.23	2.62	4	0.293	2.83
Condeixa-a-Nova	1.46	1.00	3	1.824	3.67
Covilhã	1.00	4.00	4	2.121	1.00
Estarreja	1.85	3.85	4	1.497	1.00
Figueira Castro Rodrigo	2.15	2.69	4	0.396	1.17
Figueira da Foz	1.92	3.77	4	1.394	2.58
Figueiró dos Vinhos	2.77	2.00	2	0.568	4.00

Economic implications of the different statin prescribing patterns in Central Portugal: a longitudinal analysis.

Municipality	Mean of the quartiles of the yearly distributions		quad-rant	distance	Antidepressants mean of the quartiles of the yearly distributions (DDD/1000inh/day)
	DDD/1000inh/day	cost per DDD			
Quadrant 1 High prescription High cost	Quadrant 2 High prescription Low cost		Quadrant 3 Low prescription Low cost		Quadrant 4 Low prescription High cost
Fornos Algodres	2.92	3.00	1	0.655	2.92
Fundão	1.00	3.15	4	1.636	1.00
Góis	2.77	4.00	1	1.524	3.08
Gouveia	3.38	4.00	1	1.741	2.58
Guarda	1.00	3.00	4	1.581	1.00
Idanha a Nova	2.15	2.54	4	0.348	1.17
Ílhavo	2.08	1.62	3	0.981	2.17
Leiria	1.00	2.00	3	1.581	2.08
Lousã	1.00	1.31	3	1.916	3.83
Mangualde	2.54	1.54	2	0.962	2.75
Manteigas	2.69	3.23	1	0.756	3.50
Marinha Grande	1.00	2.38	3	1.504	1.08
Mealhada	3.23	1.23	2	1.465	3.00
Meda	3.00	3.85	1	1.436	1.50
Mira	3.46	1.92	2	1.121	2.17
Miranda do Corvo	1.23	1.77	3	1.465	2.92
Montemor-o-Velho	2.77	1.92	2	0.637	2.92
Mortágua	4.00	3.46	1	1.782	4.00
Murtosa	2.00	3.46	4	1.084	1.00
Nelas	3.85	2.62	1	1.351	2.25
Oleiros	2.85	2.46	2	0.348	2.92
Oliveira de Frades	3.46	2.00	2	1.084	2.08
Oliveira do Bairro	1.77	2.62	4	0.740	2.08
Oliveira do Hospital	1.69	1.69	3	1.142	1.00
Ovar	2.08	1.23	3	1.338	2.42
Pampilhosa da Serra	3.46	2.62	1	0.968	3.83
Pedrógão Grande	4.00	2.77	1	1.524	4.00
Penacova	3.69	1.62	2	1.485	4.00
Penalva do Castelo	1.38	3.62	4	1.577	1.08
Penamacor	3.15	3.85	1	1.497	1.42
Penela	2.69	1.31	2	1.208	4.00
Pinhel	1.77	2.92	4	0.844	1.25
Pombal	2.15	2.08	3	0.547	2.25
Porto de Mós	2.46	2.46	3	0.054	2.92
Proença a Nova	3.92	2.23	2	1.448	3.58
Sabugal	1.15	3.77	4	1.850	1.00
Santa Comba Dão	4.00	1.38	2	1.869	4.00
São Pedro do Sul	3.69	2.00	2	1.293	3.50
Sátão	2.08	1.38	3	1.193	2.50
Seia	2.69	3.85	1	1.360	2.25
Sertã	1.00	1.92	3	1.607	1.00
Sever do Vouga	3.23	3.38	1	1.147	3.67
Soure	4.00	1.08	2	2.068	3.83
Tábua	3.92	1.00	2	2.068	3.75
Tondela	3.38	2.69	1	0.905	3.92
Trancoso	1.00	2.92	4	1.559	1.00
Vagos	1.54	3.15	4	1.163	1.50
Vila de Rei	2.69	2.77	1	0.331	2.58
Vila Nova de Foz Côa	2.44	3.89	4	1.390	2.00
Vila Nova de Paiva	2.23	3.46	4	0.999	4.00
Vila Nova de Poiares	3.46	2.54	1	0.962	2.33
Vila Velha Rodão	2.62	2.46	2	0.122	2.67
Viseu	1.00	1.85	3	1.636	1.33
Vouzela	3.38	3.31	1	1.198	3.83
